# Red Meat Consumption and Hypertension: An Updated Review

**DOI:** 10.1007/s11886-025-02201-2

**Published:** 2025-02-10

**Authors:** Tara S. Allen, Michael Najem, Alexis C. Wood, Danielle J. Lee, Lorena S. Pacheco, Lori B. Daniels, Matthew A. Allison

**Affiliations:** 1 Division of Preventive Medicine, Department of Family Medicine, University of California, San Diego, CA USA; 2 University of California Internal Medicine Residency Program, San Diego, CA USA; 3 USDA/ARS Children’s Nutrition Research Center, Baylor College of Medicine, Houston, TX USA; 4 Department of Nutrition, Harvard T.H. Chan School of Public Health, Boston, MA USA; 5 Division of Cardiovascular Medicine, Department of Medicine, University of California, San Diego; La Jolla, CA USA; 6 Department of Family Medicine, University of California, San Diego, CA USA

**Keywords:** Red meat, Processed red meat, Hypertension, Nutrition, Risk factor modification, Public health, Cardiovascular disease

## Abstract

**Purpose of Review:**

Hypertension (HTN) is a major risk factor for cardiovascular diseases (CVD). The global prevalence of HTN and related CVD mortality continues to rise. The development of HTN is influenced by genetic predisposition and modifiable risk factors, including diet. One area of ongoing debate is the relationship between red meat consumption and risk of HTN.

**Recent Findings:**

Processed red meat has become increasingly implicated in the pathogenesis and morbidity of HTN, though randomized control trials comparing HTN-related outcomes associated with red meat subtypes have yielded heterogenous results.

**Summary:**

This review summarizes the existing relevant literature and highlights the methodological challenges that complicate definitive conclusions, with a focus on processed versus unprocessed red meat consumption and HTN. It explores pathophysiologic mechanisms contributing to this relationship and reviews practical, evidence-based dietary guidelines that address red meat consumption to mitigate the risk of adverse HTN-related CVD outcomes.

## Introduction

Hypertension (HTN) is the leading modifiable risk factor for cardiovascular disease (CVD) in the United States (U.S.) [1, 2] and globally [3]. Despite previous advances in reducing CVD-associated morbidity and mortality, these trends have reversed in recent years [4]. In fact, the prevalence of HTN is now over 80% in U.S. adults. Even more concerning is that a 2024 National Health and Nutrition Examination Survey (NHANES) reported that (1) more younger adults are developing HTN, (2) more than half of U.S. adults with HTN were not aware of their condition, and (3) of those who were aware and took prescribed medication, 71% had uncontrolled HTN [5–8]. Indeed, U.S. health care expenses associated with HTN are projected to increase by $353 billion, representing a 220% increase from 2020 to 2050^4^. As such, measures to prevent and appropriately manage HTN are of crucial importance. The development of HTN is influenced by a combination of genetic predisposition and modifiable risk factors, including dietary patterns [9]. One area of ongoing interest is the relation between red meat consumption and HTN and related health outcomes [10–12].

With approximately 74% of U.S adults reporting red meat consumption per given day [13, 14], the U.S. is the second highest consumer of red meat globally [14]. Even after accounting for potential confounders, evidence links the dietary consumption of red meat, and especially processed red meat, with a higher risk of HTN [15–44]. When combined with an otherwise heart-healthy diet and lifestyle, though, it currently remains unclear to what degree unprocessed red meat is linked with HTN and CVD [45–50]. And, it remains unclear how changes to contemporary red meat consumption patterns may serve as an applicable intervention to prevent and decrease HTN and related complications. Some variability in the study findings is likely attributable to inconsistent definitions of red meat. Given this, the objective of this report is to briefly review and summarize the existing research on the associations and linkages between red meat and blood pressure. For the purposes of this review, we aimed to define and categorize red meat as “unprocessed red meat” or “processed red meat” and included pork-related products in the latter.

Our updated review emphasizes new research within the past 5 years, though given the dearth of focused research in this area, we have expanded the window to include further key studies. We discuss observational studies, randomized clinical trials, and delve into the currently considered and debated mechanisms regarding the associations found between red meat consumption, HTN, and CVD. Moreover, we discuss updated dietary recommendations by leading organizations regarding red meat consumption.

## Pathophysiology

Red meat consumption, and specifically processed red meat consumption, can impact several pathophysiological mechanisms contributing to the development of HTN through mediating factors such as sodium, preservatives (e.g. nitrites), and pro-inflammatory metabolites. High sodium intake has well-documented effects on the sodium-potassium ratio, increasing extracellular volume, vascular resistance, and sympathetic activity, as well as worsening endothelial inflammation, thus contributing to HTN, microvascular damage, and CVD [51–54]. Processed red meat contains approximately 400% higher sodium content compared to unprocessed red meat and is associated with higher HTN incidence [18, 27, 30, 36, 55, 56].

Nitrites and nitrates, common processed meat additives, have also demonstrated an independent but synergistic contribution (along with sodium) to BP elevation, with one study reporting a 3.1 mmHg increase in diastolic BP per increasing tertile of nitrate consumption [57, 58]. These additives have purported roles in promoting endothelial dysfunction, which is associated with BP dysregulation, and may potentially add to the higher risk of HTN observed with consumption of processed red meats relative to unprocessed red meats [59].

**Fig. 1:**
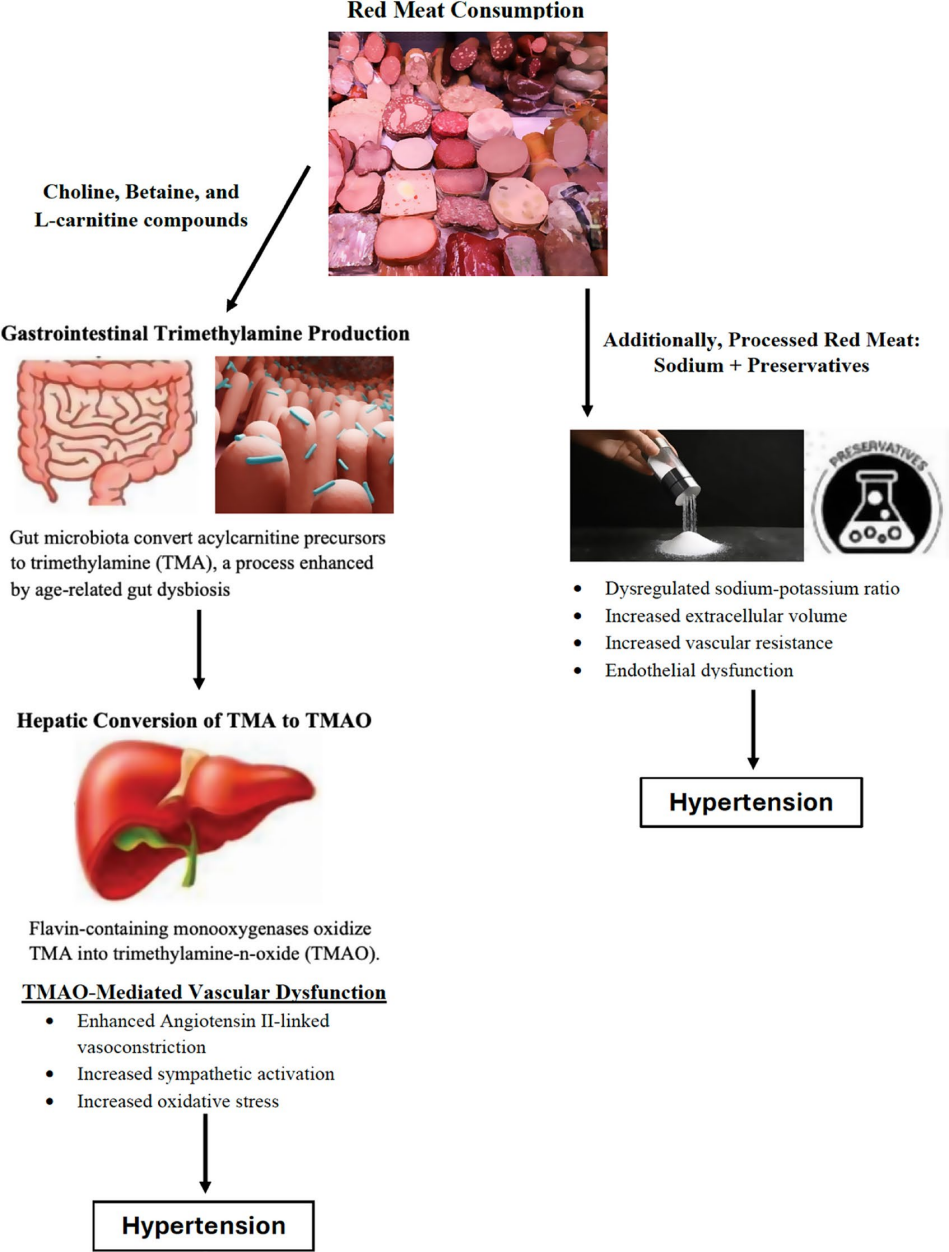
Pathophysiological mechanisms linking red meat consumption (particularly processed meat) to hypertension, including the effects of sodium, nitrite preservatives, and trimethylamine-n-oxide (TMAO). (Portions of this figure are used with permission from Oxford University Press) [10]

In addition to sodium and preservative associated effects on BP, metabolic compounds rich in all red meat, such as choline, betaine, and L-carnitine, are converted into the pathogenic metabolite trimethylamine-N-oxide (TMAO). TMAO can precipitate oxidative stress and endothelial dysfunction, both implicated in HTN and CVD [60–63]. Gut microbiota convert these precursor compounds into trimethylamine (TMA), which is shunted into the portal circulation by TMA lyases and then oxidized to TMAO by hepatic monoxygenases [64, 65]. TMAO has been implicated in angiotensin II-mediated vasoconstriction (through enhancement of intracellular calcium release), increased sympathetic activation and oxidative stress, and impairment of endothelial nitric oxide production (involved in vasodilation) (Fig. 1) [66–68].

Indeed, one U.S.-based cohort study with a median 12.5 year follow-up isolated a specific, significant mediatory effect of TMAO-related metabolites on red meat-associated development of atherosclerotic CVD (ASCVD) [69]. A meta-analysis of 15 studies and 15,498 subjects found an increased relative risk of HTN with higher circulating TMAO levels (1.14, 95% CI 1.08–1.20), and although the RR is relatively small for such a large sample size, this relationship was dose-dependent with a 1.014% increase in HTN risk for every increase of TMAO level by 1 µ mol/L [70]. Furthermore, age-related changes in gut microbiota favor microbiologic species that induce higher production of TMAO, correlating with the increase in endothelial dysfunction, subsequent atherosclerosis, and CVD associated with aging [61, 62, 64, 67].

The relationship of unprocessed meat to HTN is less clear. Some large scale meta-analyses show no association, others show associations only in certain sub-populations, and of those that show an association, many do not control for intake of processed meat in the analyses or meta-analyses [18, 27, 30, 31, 36, 55, 56]. While several studies implicate unprocessed red meat with the aforementioned deleterious effects on HTN and cardiovascular health, the increased sodium and preservative content of processed meat highlight the need for specialized attention to its HTN-related pathogenicity [27, 58]. Therefore, further population-based studies are needed to isolate and differentiate the impact of the subtypes of red meat intake on HTN and CVD.

## Observational Studies

Longitudinal cohort studies and meta-analyses link red meat consumption to a host of adverse outcomes including stroke, heart failure, CVD-related and all-cause mortality, with HTN cited as the likely mediating factor [30, 42, 44, 69, 71]. Studies have also demonstrated dose dependency for this association. Specifically, data from a 2021 NHANES report of 31,314 U.S. adults found that compared with the lowest level of red meat intake, those in the fourth and fifth quintiles had a 29% and 39% higher odds of HTN, respectively (p-trend < 0.01).^15^ Conversely, substituting one serving of red meat per day with either poultry, fish, eggs, dairy, or plant-based protein was associated with an 8–15% lower odds of HTN [15]. A meta-analysis of cohort studies demonstrated a positive association between red meat intake and HTN risk with a pooled multivariable-adjusted relative risk (RR) of 1.22 (*p* < 0.01).^36^ Among the included studies, the Coronary Artery Risk Development in Young Adults (CARDIA) study followed participants for 15 years and found that red meat consumption of ≥ 1–2 times/day was associated with a 20–40% higher risk of HTN compared to < 0.6 times/day; and there was a dose-dependent relation across increasing quintiles of red meat consumption (p-trend = 0.004).^40^

There is a stronger and more consistent association between processed meat consumption and HTN and CVD outcomes, compared to unprocessed red meat [18, 27, 30, 41–43, 55, 71, 72]. One study observed a significantly higher risk of HTN in those who consumed ≥ 5 versus < 1 serving/week of processed red meat (50 g serving, 17% higher risk), but not with unprocessed red meat [41]. Another study of participants with HTN on hemodialysis found that red meat, and particularly processed red meat, was significantly associated with more difficult to control BP and HTN [19]. Yet another systematic review with meta-analysis found that each additional 100 g/day of overall red meat consumption conferred a 14% higher risk of HTN (P-heterogeneity < 0.001; *N* = 7) but when just processed red meat was considered, only 50 additional g/day conferred a 12% higher risk of HTN (P-heterogeneity < 0.001; *N* = 4) [73].

Although the previously noted studies assessed the association between red meat consumption and BP or HTN outcomes, many other studies focused on the association between red meat consumption and CVD or mortality. In these studies, the associations with adverse outcomes tended to be stronger for processed than for unprocessed red meat. For example, an analysis of over 100,000 individuals from 21 countries found significant associations between high consumption of processed meat and mortality (HR: 1.51; 95% CI: 1.08–2.10; p-trend = 0.009) as well as major CVD (HR: 1.46; 95% CI: 1.08–1.98; p-trend = 0.004), but no significant associations with unprocessed red meat intake [42]. Another study found a significant risk for all stroke subtypes when processed red meat consumption exceeded 0 g/day and unprocessed red meat consumption exceeded 70 g/day.^44^ A recent large, community-based cohort study reported a 22% higher risk of atherosclerotic CVD per each additional 1.1 serving/day of overall red meat, with processed red meat consumption associated with higher risk, specifically due to measured gut microbiome metabolite changes [69]. A meta-analysis assessing over 150,000 deaths found pooled relative risks of 1.15 (95% CI 1.11–1.19) and 1.15 (95% CI 1.07–1.24) for all-cause mortality and CVD-associated mortality, respectively, with an increase of just one serving of processed meat per day (100 g for total and unprocessed red meat; 50 g for processed meat) [28]. Some studies suggest the associations are mediated by HTN given an attenuation in effect size with relevant sensitivity analyses accounting for HTN. Also, they found that based on the dose effect seen, some limitation of red meat consumption, especially that of processed red meat, may lower risk of CVD [74–76]. 

## Randomized Controlled Trials (RCTs)

Consistently, RCTs that assess plant-based dietary interventions demonstrate improved BP, HTN, and CVD outcomes [77, 78]. While long-term observational studies have demonstrated associations between red meat consumption and HTN, BP control, and CVD outcomes, data from RCTs remain overall inconclusive. This is likely due in part to inconsistent trial methods, including difference patient populations, length of enrollment and follow-up for outcomes, measures or available data on adherence, and an overall dearth of comparable trials [11, 49, 50, 79–81]. Additionally, the design of these trials, such as crossover or parallel, run-in, and non-blinded, can influence the interpretation of results. Also, few trials have directly investigated the differences between processed versus unprocessed red meat consumption with HTN or clinically relevant BP-mediated outcomes. These factors collectively complicate the ability to draw definitive, detailed conclusions about the direct causal impact of red meat consumption on HTN risk.

Although most trials on red meat consumption and health outcomes have predominantly focused on lipids as risk factors of CVD [45, 49, 50], there are a few RCTs and meta-analyses of RCTs with a primary outcome of BP measures [46, 80, 81]. Two meta-analyses of RCTs evaluated BP outcomes related to overall red meat consumption of ≥0.5 versus < 0.5 servings/day and concluded that there was no significant difference [49, 50]. They also found no difference in blood lipids or lipoproteins. However, limitations of these analyses included heterogeneity among the individual studies and lack of data regarding processed versus unprocessed red meat as well as lack of data on adherence to the trial protocols. Other trials demonstrate the importance of specific red meat characteristics, such as the type of red meat (processed versus unprocessed), degree of saturated fat, preparation methods, and combination of other components of the diet, in regards to BP and HTN outcomes [46, 47, 82]. 

Adding to the lack of certainty, evidence from RCTs does not currently provide conclusive evidence of a reduction in CVD risk factors, including HTN, when either processed or unprocessed meat intake is reduced or eliminated, though most trials were small and of limited duration [50, 79]. A recent meta-analysis of twenty RCTs evaluating the effects of unprocessed beef intake on CVD risk factors including HTN concluded that daily unprocessed beef consumption did not significantly impact BP (neither diastolic nor systolic), circulating lipoprotein lipid and apolipoprotein measures, although a small lowering of LDL-C levels was seen with elimination of unprocessed meat [50, 79]. There are important and significant limitations to this presented RCT evidence, since the trials were highly heterogeneous with regards to the compositions of dietary interventions, length of the intervention, follow-up times, and other critical overall trial approaches, making it difficult to establish appropriate comparisons and effectively draw conclusions.

## Current Guidelines and Recommendations on Red Meat Consumption

Diet has been firmly established as a significant modifiable environmental factor and cornerstone in HTN treatment [83, 84]. Vegetarian diets are associated with reduced HTN incidence, and even when omnivore diets meet high plant-based standards, the association is not fully attenuated [83, 85, 86]. These effects extend beyond HTN to HTN-related comorbidities, as a recent study with 30-year follow-up found significantly lower rates of coronary artery disease and total CVD in participants with the highest plant to animal protein balance to those with the lowest in a dose-responsive relationship [87]. Although red meat can be a major source of valuable nutrients, such as protein, iron, zinc, and vitamin B12 [88, 89], epidemiological studies supporting an association between red meat and HTN have led to the many dietary guidelines for health recommending that red meat is minimized or removed from the diet. It may be particularly important to minimize or remove processed red meat.

Several North American public health guidelines currently recommend limiting red and processed meat consumption, including national dietary guidelines from the US, Canada, and Mexico [13]. The European EAT-Lancet commission guidelines and the Harvard School of Public Health recommend against any processed meat intake to promote health [13, 90]. The Dietary Guidelines for Americans (DGA) takes a more nuanced approach to the inclusion of meat products in a diet designed to promote health and prevent chronic disease. The current DGA (2020–2025) notes both that dietary patterns linked with positive health outcomes, including lower risk of CVD, typically include relatively lower red and processed meat consumption, and that diets higher in lean meat are comparatively associated with more positive health outcomes, ultimately recommending only that most intake of meat should be in lean and unprocessed forms versus processed forms [91, 92]. Similarly, and noting the uncertainty in the literature on overall red meat versus processed red meat versus unprocessed red meat described above, the National Lipid Association and the American Heart Association (AHA) both recommend diets focused more on lean, unprocessed meat intake [93, 94], although the AHA more specifically adds the recommendation to limit red meat intake to less than 50–100 g per day, even if lean and unprocessed, in their “Life’s Essential 8” dietary component metric [95].

Worldwide, a consensus on red meat in a healthy diet has yet to be reached, though most recommend limiting any red meat intake to 500 g per week or even less. For example, the National Health Service of UK through their 2023 Eatwell Guide recommends limited processed meat consumption and specifically encourages adults to reduce their consumption of red or processed meat to < 70 g per day [96]. The Australian Dietary Guidelines, last updated in 2019, encourages a moderate consumption of red meat, recommending lean forms over processed and a maximum intake of 455 g per week (65 g per day) of lean meats to reduce health risks linked with meat consumption and encourage dietary variety for Australian adults [97]. The Nordic Nutrition Recommendations (NNR) 2023 recommend limited intake of red meat and minimal (as low as possible) intake of processed red meat [98]. The recommended dietary changes in the updated 2020 Eating and Activity Guidelines for New Zealand Adults is to limit processed and red meat, eating less than 500 g of cooked red meat per week [99]. The 2015 Dutch Dietary Guidelines and the 2023 updates specifically targeted to patients with established CVD recommend limiting meat consumption, especially red or processed meat [100]. Taken together, most guidelines recommend limiting red meat intake to at most a moderate daily amount of unprocessed forms, although there are notable exceptions that may be influenced by numerous factors beyond evidence-based research.

From a more focused public health perspective, certain groups may particularly benefit from reducing red meat consumption. Among U.S populations, overall and processed red meat consumption is significantly higher among adult men, those of lower educational and socioeconomic statuses (SES), and specific racial and ethnic groups, particularly those who identity as Black [13, 18–20, 101]. Such differences in dietary patterns may perpetuate HTN and CVD-related health disparities and highlight the need for more personalized nutritional guidance related to red meat consumption, which is not currently part of national guidelines.

## Conclusions

Results from pathophysiology studies suggest a potential deleterious effect of red meat consumption, especially that of processed red meat, that may be mediated by several different mechanisms. Data from observational studies provide further support for these associations. However, clinical trial research on this topic is heterogeneous and limited, and differences in the associations between processed and unprocessed red meat with adverse outcomes is less clear. This highlights the complexity of dietary interventions and the need for more nuanced research, particularly longer-term RCTs, to identify the specific types and amounts of meat that pose the greatest risk. Furthermore, public health strategies to reduce HTN-related complications must include specific guidelines on how red meat consumption, especially that of processed red meat, is a relevant component in the overall dietary health equation. Comprehensive dietary and lifestyle modifications that address other factors such as overall sodium and caloric intake, physical activity, and sedentary behavior are also important. As we continue to examine the impact of red meat on HTN, future studies should focus on clarifying the mechanisms underlying these associations and assessing the effectiveness of targeted dietary changes in diverse populations. As red meat consumption currently is a notable aspect of the U.S. diet, it is important to explore how dietary patterns, particularly the consumption of processed red meat, may contribute to the rising rates of HTN and poor HTN control.

## Key References

1. Schwingshackl L, Schwedhelm C, Hoffmann G, et al. Food Groups and Risk of Hypertension: A Systematic Review and Dose-Response Meta-Analysis of Prospective Studies. *Adv Nutr.* 2017;8(6):793–803.

This meta-analysis of 28 prospective studies demonstrated a statistically signifcant and dose-responsive relationship between red meat intake and HTN, with a more potent association identified with processed red meat.

2. Han JM, Guo L, Chen XH, Xie Q, Song XY, Ma YL. Relationship between trimethylamine N-oxide and the risk of hypertension in patients with cardiovascular disease: A meta-analysis and dose-response relationship analysis. *Medicine (Baltimore).* 2024;103(1):e36784.

This meta-analysis demonstrates a significant association between trimethylamine-N-oxide (a known metabolite of compounds abundant in red meat) and hypertension in a dose-dependent manner.

3. Guasch-Ferre M, Satija A, Blondin SA, et al. Meta-Analysis of Randomized Controlled Trials of Red Meat Consumption in Comparison With Various Comparison Diets on Cardiovascular Risk Factors. *Circulation.* 2019;139(15):1828–1845.

This meta-analysis of randomized control trials demonstrated no difference in effect on blood pressure between higher red meat containing diets (> 0.5 servings/day) and lower red meat-containing diets (< 0.5 servings/day), highlighting the uncertainty of evidence in randomized studies as compared to prospective studies.

## Data Availability

No datasets were generated or analysed during the current study.

## References

[1] Fuchs FD, Whelton PK. High blood pressure and Cardiovascular Disease. Hypertension. 2020;75(2):285–92. 31865786 10.1161/HYPERTENSIONAHA.119.14240 PMC10243231

[2] Yano Y, Reis JP, Colangelo LA, et al. Association of blood pressure classification in young adults using the 2017 American College of Cardiology/American Heart Association blood pressure Guideline with Cardiovascular events later in Life. JAMA. 2018;320(17):1774–82. 30398601 10.1001/jama.2018.13551 PMC6248102

[3] Vaduganathan M, Mensah GA, Turco JV, Fuster V, Roth GA. The Global Burden of Cardiovascular diseases and Risk: a compass for Future Health. J Am Coll Cardiol. 2022;80(25):2361–71. 36368511 10.1016/j.jacc.2022.11.005

[4] Joynt Maddox KE, Elkind MSV, Aparicio HJ, et al. Forecasting the Burden of Cardiovascular Disease and Stroke in the United States through 2050-Prevalence of risk factors and disease: a Presidential Advisory from the American Heart Association. Circulation. 2024;150(4):e65–88. 38832505 10.1161/CIR.0000000000001256

[5] Hypertension Cascade: Hypertension Prevalence, Treatment and Control Estimates Among US Adults Aged 18 Years and Older Applying the Criteria From the American College of Cardiology and American Heart Association’s 2017 Hypertension Guideline—NHANES 2017–2020. Centers for Disease Control and Prevention (CDC). https://millionhearts.hhs.gov/data-reports/hypertension-prevalence.html. Published 2023. Accessed November 7, 2024, 2024.

[6] Ostchega YFC, Nwankwo T, Nguyen DT. Hypertension prevalence among adults aged 18 and over: United States, 2017–2018. National Center for Health Statistics NCHS Data Brief, no 364 Web site. Published 2020. Accessed November 7, 2024, 2024. 32487290

[7] Fryar CDKB, Carroll MD, Afful J. Hypertension prevalence, awareness, treatment, and control among adults age 18 and older: United States, August 2021–August 2023. National Center for Health Statistics. NCHS Data Brief, no 511 Web site. Published 2024. Accessed. 10.15620/cdc/164016 PMC13655856 40085792

[8] Richardson LC, Vaughan AS, Wright JS, Coronado F. Examining the Hypertension Control Cascade in adults with uncontrolled hypertension in the US. JAMA Netw Open. 2024;7(9):e2431997. 39259543 10.1001/jamanetworkopen.2024.31997 PMC11391330

[9] Mukherjee D, Whayne TF. Editorial (thematic issue: sex, Environment, Physical Activity, Diet, and obesity in Cardiovascular Disease Risk). Curr Vasc Pharmacol. 2016;14(5):406–8. 27809757 10.2174/138955751617161006172905

[10] Shi W, Huang X, Schooling CM, Zhao JV. Red meat consumption, cardiovascular diseases, and diabetes: a systematic review and meta-analysis. Eur Heart J. 2023;44(28):2626–35. 37264855 10.1093/eurheartj/ehad336

[11] Allen TS, Bhatia HS, Wood AC, Momin SR, Allison MA. State-of-the-art review: evidence on Red Meat Consumption and Hypertension outcomes. Am J Hypertens. 2022;35(8):679–87. 35561332 10.1093/ajh/hpac064 PMC10060708

[12] Liu D, Shi Q, Cheng G, Huang Q, Li S. Worldwide burden attributable to diet high in red meat from 1990 to 2019. Arch Med Sci. 2023;19(1):1–15. 36817670 10.5114/aoms/156017 PMC9897098

[13] Frank SM, Jaacks LM, Batis C, Vanderlee L, Taillie LS. Patterns of Red and processed meat consumption across North America: a nationally Representative Cross-sectional comparison of Dietary recalls from Canada, Mexico, and the United States. Int J Environ Res Public Health 2021;18(1). 10.3390/ijerph18010357 PMC7796493 33466518

[14] Development OfEC-oa. Meat Consumption https://data.oecd.org/agroutput/meat-consumption.htm. Published 2021. Accessed Feburary 13, 2022.

[15] Ba DM, Gao X, Chinchilli VM, Liao D, Richie JP Jr., Al-Shaar L. Red and processed meat consumption and food insecurity are associated with hypertension; analysis of the national health and nutrition examination survey data, 2003–2016. J Hypertens. 2022;40(3):553–560. 10.1097/HJH.0000000000003048 34784309

[16] Atalic B, Toth J, Atalic V, Radanovic D, Miskulin M, Lucin A. Red and processed meat and cardiovascular risk factors. Acta Med Croatica. 2013;67(3):211–8. 25007430

[17] Masala G, Bendinelli B, Versari D, et al. Anthropometric and dietary determinants of blood pressure in over 7000 Mediterranean women: the European prospective investigation into Cancer and Nutrition-Florence cohort. J Hypertens. 2008;26(11):2112–20. 18854749 10.1097/HJH.0b013e32830ef75c

[18] Oude Griep LM, Seferidi P, Stamler J, et al. Relation of unprocessed, processed red meat and poultry consumption to blood pressure in east Asian and western adults. J Hypertens. 2016;34(9):1721–9. 27379533 10.1097/HJH.0000000000001008 PMC6524524

[19] Wu PY, Yang SH, Wong TC, et al. Association of Processed Meat Intake with Hypertension Risk in Hemodialysis patients: a cross-sectional study. PLoS ONE. 2015;10(10):e0141917. 26517837 10.1371/journal.pone.0141917 PMC4627724

[20] Tzoulaki I, Brown IJ, Chan Q, et al. Relation of iron and red meat intake to blood pressure: cross sectional epidemiological study. BMJ. 2008;337:a258. 18632704 10.1136/bmj.a258 PMC2658466

[21] Zhang J, Hayden K, Jackson R, Schutte R. Association of red and processed meat consumption with cardiovascular morbidity and mortality in participants with and without obesity: a prospective cohort study. Clin Nutr. 2021;40(5):3643–9. 33413912 10.1016/j.clnu.2020.12.030

[22] Sheehy S, Palmer JR, Rosenberg L. High consumption of Red Meat is Associated with excess mortality among African-American women. J Nutr. 2020;150(12):3249–58. 33024986 10.1093/jn/nxaa282 PMC7726124

[23] Zheng Y, Li Y, Satija A, et al. Association of changes in red meat consumption with total and cause specific mortality among US women and men: two prospective cohort studies. BMJ. 2019;365:l2110. 31189526 10.1136/bmj.l2110 PMC6559336

[24] Alshahrani SM, Fraser GE, Sabate J et al. Red and processed meat and mortality in a low meat Intake Population. Nutrients 2019;11(3). 10.3390/nu11030622 PMC6470727 30875776

[25] Bellavia A, Stilling F, Wolk A. High red meat intake and all-cause cardiovascular and cancer mortality: is the risk modified by fruit and vegetable intake? Am J Clin Nutr. 2016;104(4):1137–43. 27557655 10.3945/ajcn.116.135335

[26] Pan A, Sun Q, Bernstein AM, et al. Red meat consumption and mortality: results from 2 prospective cohort studies. Arch Intern Med. 2012;172(7):555–63. 22412075 10.1001/archinternmed.2011.2287 PMC3712342

[27] Zhong VW, Van Horn L, Greenland P, et al. Associations of processed meat, unprocessed Red Meat, Poultry, or Fish Intake With Incident Cardiovascular Disease and all-cause mortality. JAMA Intern Med. 2020;180(4):503–12. 32011623 10.1001/jamainternmed.2019.6969 PMC7042891

[28] Wang X, Lin X, Ouyang YY, et al. Red and processed meat consumption and mortality: dose-response meta-analysis of prospective cohort studies. Public Health Nutr. 2016;19(5):893–905. 26143683 10.1017/S1368980015002062 PMC10270853

[29] Bechthold A, Boeing H, Schwedhelm C, et al. Food groups and risk of coronary heart disease, stroke and heart failure: a systematic review and dose-response meta-analysis of prospective studies. Crit Rev Food Sci Nutr. 2019;59(7):1071–90. 29039970 10.1080/10408398.2017.1392288

[30] Abete I, Romaguera D, Vieira AR, Lopez de Munain A, Norat T. Association between total, processed, red and white meat consumption and all-cause, CVD and IHD mortality: a meta-analysis of cohort studies. Br J Nutr. 2014;112(5):762–75. 24932617 10.1017/S000711451400124X

[31] Micha R, Michas G, Lajous M, Mozaffarian D. Processing of meats and cardiovascular risk: time to focus on preservatives. BMC Med. 2013;11:136. 23701737 10.1186/1741-7015-11-136 PMC3680013

[32] Micha R, Shulkin ML, Penalvo JL, et al. Etiologic effects and optimal intakes of foods and nutrients for risk of cardiovascular diseases and diabetes: systematic reviews and meta-analyses from the Nutrition and Chronic diseases Expert Group (NutriCoDE). PLoS ONE. 2017;12(4):e0175149. 28448503 10.1371/journal.pone.0175149 PMC5407851

[33] Zhuang P, Jiao J, Wu F, Mao L, Zhang Y. Associations of meat consumption and changes with all-cause mortality in hypertensive patients during 11.4-year follow-up: findings from a population-based nationwide cohort. Clin Nutr. 2021;40(3):1077–84. 32741682 10.1016/j.clnu.2020.06.040

[34] Wang L, Manson JE, Buring JE, Sesso HD. Meat intake and the risk of hypertension in middle-aged and older women. J Hypertens. 2008;26(2):215–22. 18192834 10.1097/HJH.0b013e3282f283dc

[35] Borgi L, Curhan GC, Willett WC, Hu FB, Satija A, Forman JP. Long-term intake of animal flesh and risk of developing hypertension in three prospective cohort studies. J Hypertens. 2015;33(11):2231–8. 26237562 10.1097/HJH.0000000000000722 PMC4797063

[36] Zhang Y, Zhang DZ. Red meat, poultry, and egg consumption with the risk of hypertension: a meta-analysis of prospective cohort studies. J Hum Hypertens. 2018;32(7):507–17. 29725070 10.1038/s41371-018-0068-8

[37] Miura K, Greenland P, Stamler J, Liu K, Daviglus ML, Nakagawa H. Relation of vegetable, fruit, and meat intake to 7-year blood pressure change in middle-aged men: the Chicago Western Electric Study. Am J Epidemiol. 2004;159(6):572–80. 15003961 10.1093/aje/kwh085

[38] Ascherio A, Hennekens C, Willett WC, et al. Prospective study of nutritional factors, blood pressure, and hypertension among US women. Hypertension. 1996;27(5):1065–72. 8621198 10.1161/01.hyp.27.5.1065

[39] Lelong H, Blacher J, Baudry J, et al. Individual and Combined effects of Dietary factors on risk of Incident Hypertension: prospective analysis from the NutriNet-Sante Cohort. Hypertension. 2017;70(4):712–20. 28760943 10.1161/HYPERTENSIONAHA.117.09622

[40] Steffen LM, Kroenke CH, Yu X, et al. Associations of plant food, dairy product, and meat intakes with 15-y incidence of elevated blood pressure in young black and white adults: the coronary artery Risk Development in Young adults (CARDIA) study. Am J Clin Nutr. 2005;82(6):1169–77. quiz 1363– 1164. 16332648 10.1093/ajcn/82.6.1169

[41] Lajous M, Bijon A, Fagherazzi G, Rossignol E, Boutron-Ruault MC, Clavel-Chapelon F. Processed and unprocessed red meat consumption and hypertension in women. Am J Clin Nutr. 2014;100(3):948–52. 25080454 10.3945/ajcn.113.080598

[42] Iqbal R, Dehghan M, Mente A, et al. Associations of unprocessed and processed meat intake with mortality and cardiovascular disease in 21 countries [Prospective Urban Rural Epidemiology (PURE) Study]: a prospective cohort study. Am J Clin Nutr. 2021;114(3):1049–58. 33787869 10.1093/ajcn/nqaa448

[43] Micha R, Wallace SK, Mozaffarian D. Red and processed meat consumption and risk of incident coronary heart disease, stroke, and diabetes mellitus: a systematic review and meta-analysis. Circulation. 2010;121(21):2271–83. 20479151 10.1161/CIRCULATIONAHA.109.924977 PMC2885952

[44] Yang C, Pan L, Sun C, Xi Y, Wang L, Li D. Red Meat Consumption and the risk of stroke: a dose-response Meta-analysis of prospective cohort studies. J Stroke Cerebrovasc Dis. 2016;25(5):1177–86. 26935118 10.1016/j.jstrokecerebrovasdis.2016.01.040

[45] Appel LJ, Sacks FM, Carey VJ, et al. Effects of protein, monounsaturated fat, and carbohydrate intake on blood pressure and serum lipids: results of the OmniHeart randomized trial. JAMA. 2005;294(19):2455–64. 16287956 10.1001/jama.294.19.2455

[46] Nowson CA, Wattanapenpaiboon N, Pachett A. Low-sodium Dietary approaches to stop hypertension-type diet including lean red meat lowers blood pressure in postmenopausal women. Nutr Res. 2009;29(1):8–18. 19185772 10.1016/j.nutres.2008.12.002

[47] Wang Z, Huang Q, Wang L et al. Moderate Intake of Lean Red Meat Was Associated with Lower risk of elevated blood pressure in Chinese women: results from the China Health and Nutrition Survey, 1991–2015. Nutrients 2020;12(5). 10.3390/nu12051369 PMC7284636 32403294

[48] Sayer RD, Wright AJ, Chen N, Campbell WW. Dietary approaches to Stop Hypertension diet retains effectiveness to reduce blood pressure when lean pork is substituted for chicken and fish as the predominant source of protein. Am J Clin Nutr. 2015;102(2):302–8. 26063693 10.3945/ajcn.115.111757 PMC4515871

[49] Guasch-Ferre M, Satija A, Blondin SA, et al. Meta-analysis of Randomized controlled trials of Red Meat Consumption in Comparison with various comparison diets on Cardiovascular Risk factors. Circulation. 2019;139(15):1828–45. 30958719 10.1161/CIRCULATIONAHA.118.035225

[50] O’Connor LE, Kim JE, Campbell WW. Total red meat intake of >/=0.5 servings/d does not negatively influence cardiovascular disease risk factors: a systemically searched meta-analysis of randomized controlled trials. Am J Clin Nutr. 2017;105(1):57–69. 27881394 10.3945/ajcn.116.142521 PMC5183733

[51] Marketou ME, Maragkoudakis S, Anastasiou I, et al. Salt-induced effects on microvascular function: a critical factor in hypertension mediated organ damage. J Clin Hypertens (Greenwich). 2019;21(6):749–57. 31002481 10.1111/jch.13535 PMC8030330

[52] Grillo A, Salvi L, Coruzzi P, Salvi P, Parati G. Sodium intake and hypertension. Nutrients 2019;11(9). 10.3390/nu11091970 PMC6770596 31438636

[53] Kurtz TW, DiCarlo SE, Pravenec M, Morris RC. Jr. The American Heart Association Scientific Statement on salt sensitivity of blood pressure: prompting consideration of alternative conceptual frameworks for the pathogenesis of salt sensitivity? J Hypertens. 2017;35(11):2214–25. 28650918 10.1097/HJH.0000000000001458

[54] Mente A, O’Donnell M, Rangarajan S, et al. Associations of urinary sodium excretion with cardiovascular events in individuals with and without hypertension: a pooled analysis of data from four studies. Lancet. 2016;388(10043):465–75. 27216139 10.1016/S0140-6736(16)30467-6

[55] Mendes MIF, Mendonca RD, Aprelini CMO, Molina M. Consumption of processed meat but not red meat is associated with the incidence of hypertension: ELSA-Brasil cohort. Nutrition. 2024;127:112529. 39154548 10.1016/j.nut.2024.112529

[56] Batubo NP, Moore JB, Zulyniak MA. Dietary factors and hypertension risk in West Africa: a systematic review and meta-analysis of observational studies. J Hypertens. 2023;41(9):1376–88. 37432889 10.1097/HJH.0000000000003499 PMC10399948

[57] Kotopoulou S, Zampelas A, Magriplis E. Nitrite and nitrate intake from processed meat is associated with elevated diastolic blood pressure (DBP). Clin Nutr. 2023;42(5):784–92. 37023524 10.1016/j.clnu.2023.03.015

[58] Srour B, Chazelas E, Fezeu LK, et al. Nitrites, nitrates, and Cardiovascular outcomes: are we living La Vie en Rose with Pink processed meats? J Am Heart Assoc. 2022;11(24):e027627. 36533633 10.1161/JAHA.122.027627 PMC9798789

[59] Kleinbongard P, Dejam A, Lauer T, et al. Plasma nitrite concentrations reflect the degree of endothelial dysfunction in humans. Free Radic Biol Med. 2006;40(2):295–302. 16413411 10.1016/j.freeradbiomed.2005.08.025

[60] Brunt VE, Gioscia-Ryan RA, Casso AG, et al. Trimethylamine-N-Oxide promotes Age-related vascular oxidative stress and endothelial dysfunction in mice and healthy humans. Hypertension. 2020;76(1):101–12. 32520619 10.1161/HYPERTENSIONAHA.120.14759 PMC7295014

[61] Beckman JA, Shibao CA. Trimethylamine-N-Oxide, more red meat for the vascular scientists. Hypertension. 2020;76(1):40–1. 32520618 10.1161/HYPERTENSIONAHA.120.14857 PMC7310574

[62] Koeth RA, Wang Z, Levison BS, et al. Intestinal microbiota metabolism of L-carnitine, a nutrient in red meat, promotes atherosclerosis. Nat Med. 2013;19(5):576–85. 23563705 10.1038/nm.3145 PMC3650111

[63] O’Sullivan A, Gibney MJ, Brennan L. Dietary intake patterns are reflected in metabolomic profiles: potential role in dietary assessment studies. Am J Clin Nutr. 2011;93(2):314–21. 21177801 10.3945/ajcn.110.000950

[64] Thogersen R, Rasmussen MK, Sundekilde UK et al. Background Diet influences TMAO Concentrations Associated with Red Meat Intake without influencing apparent hepatic TMAO-Related activity in a Porcine Model. Metabolites 2020;10(2). 10.3390/metabo10020057 PMC7074160 32041174

[65] Tang WH, Wang Z, Levison BS, et al. Intestinal microbial metabolism of phosphatidylcholine and cardiovascular risk. N Engl J Med. 2013;368(17):1575–84. 23614584 10.1056/NEJMoa1109400 PMC3701945

[66] Jiang S, Shui Y, Cui Y, et al. Gut microbiota dependent trimethylamine N-oxide aggravates angiotensin II-induced hypertension. Redox Biol. 2021;46:102115. 34474396 10.1016/j.redox.2021.102115 PMC8408632

[67] Ke Y, Li D, Zhao M, et al. Gut flora-dependent metabolite Trimethylamine-N-oxide accelerates endothelial cell senescence and vascular aging through oxidative stress. Free Radic Biol Med. 2018;116:88–100. 29325896 10.1016/j.freeradbiomed.2018.01.007

[68] Liu G, Cheng J, Zhang T, et al. Inhibition of Microbiota-dependent trimethylamine N-Oxide production ameliorates high Salt Diet-Induced sympathetic excitation and hypertension in rats by attenuating Central Neuroinflammation and oxidative stress. Front Pharmacol. 2022;13:856914. 35359866 10.3389/fphar.2022.856914 PMC8961329

[69] Wang M, Wang Z, Lee Y, et al. Dietary meat, trimethylamine N-Oxide-related metabolites, and Incident Cardiovascular Disease among older adults: the Cardiovascular Health Study. Arterioscler Thromb Vasc Biol. 2022;42(9):e273–88. 35912635 10.1161/ATVBAHA.121.316533 PMC9420768

[70] Han JM, Guo L, Chen XH, Xie Q, Song XY, Ma YL. Relationship between trimethylamine N-oxide and the risk of hypertension in patients with cardiovascular disease: a meta-analysis and dose-response relationship analysis. Med (Baltim). 2024;103(1):e36784. 10.1097/MD.0000000000036784 PMC10766215 38181288

[71] Chen GC, Lv DB, Pang Z, Liu QF. Red and processed meat consumption and risk of stroke: a meta-analysis of prospective cohort studies. Eur J Clin Nutr. 2013;67(1):91–5. 23169473 10.1038/ejcn.2012.180

[72] Kaluza J, Akesson A, Wolk A. Long-term processed and unprocessed red meat consumption and risk of heart failure: a prospective cohort study of women. Int J Cardiol. 2015;193:42–6. 26005173 10.1016/j.ijcard.2015.05.044

[73] Schwingshackl L, Schwedhelm C, Hoffmann G, et al. Food groups and Risk of Hypertension: a systematic review and dose-response Meta-analysis of prospective studies. Adv Nutr. 2017;8(6):793–803. 29141965 10.3945/an.117.017178 PMC5683007

[74] Al-Shaar L, Satija A, Wang DD, et al. Red meat intake and risk of coronary heart disease among US men: prospective cohort study. BMJ. 2020;371:m4141. 33268459 10.1136/bmj.m4141 PMC8030119

[75] Alshahrani SM, Fraser GE, Sabaté J, et al. Red and processed meat and mortality in a low meat Intake Population. Nutrients. 2019;11(3):622. 30875776 10.3390/nu11030622 PMC6470727

[76] Dybvik JS, Svendsen M, Aune D. Vegetarian and vegan diets and the risk of cardiovascular disease, ischemic heart disease and stroke: a systematic review and meta-analysis of prospective cohort studies. Eur J Nutr. 2023;62(1):51–69. 36030329 10.1007/s00394-022-02942-8 PMC9899747

[77] Tome-Carneiro J, Visioli F. Plant-based diets reduce blood pressure: a systematic review of recent evidence. Curr Hypertens Rep. 2023;25(7):127–50. 37178356 10.1007/s11906-023-01243-7 PMC10224875

[78] Toh DWK, Fu AS, Mehta KA, Lam NYL, Haldar S, Henry CJ. Plant-based meat analogs and their effects on Cardiometabolic Health: an 8-Week randomized controlled trial comparing plant-based meat analogs with their corresponding animal-based foods. Am J Clin Nutr. 2024;119(6):1405–16. 38599522 10.1016/j.ajcnut.2024.04.006

[79] Sanders LM, Wilcox ML, Maki KC. Red meat consumption and risk factors for type 2 diabetes: a systematic review and meta-analysis of randomized controlled trials. Eur J Clin Nutr. 2023;77(2):156–65. 35513448 10.1038/s41430-022-01150-1 PMC9908545

[80] O’Connor LE, Paddon-Jones D, Wright AJ, Campbell WW. A Mediterranean-style eating pattern with lean, unprocessed red meat has cardiometabolic benefits for adults who are overweight or obese in a randomized, crossover, controlled feeding trial. Am J Clin Nutr. 2018;108(1):33–40. 29901710 10.1093/ajcn/nqy075 PMC6600057

[81] Appel LJ, Moore TJ, Obarzanek E, et al. A clinical trial of the effects of dietary patterns on blood pressure. DASH Collaborative Research Group. N Engl J Med. 1997;336(16):1117–24. 9099655 10.1056/NEJM199704173361601

[82] Montoro-Garcia S, Velasco-Soria A, Mora L et al. Beneficial impact of pork dry-cured Ham Consumption on blood pressure and cardiometabolic markers in individuals with Cardiovascular Risk. Nutrients 2022;14(2). 10.3390/nu14020298 PMC8777827 35057479

[83] Yokoyama Y, Nishimura K, Barnard ND, et al. Vegetarian diets and blood pressure: a meta-analysis. JAMA Intern Med. 2014;174(4):577–87. 24566947 10.1001/jamainternmed.2013.14547

[84] Chobanian AV, Bakris GL, Black HR, et al. Seventh report of the Joint National Committee on Prevention, detection, evaluation, and treatment of high blood pressure. Hypertension. 2003;42(6):1206–52. 14656957 10.1161/01.HYP.0000107251.49515.c2

[85] Appleby PN, Davey GK, Key TJ. Hypertension and blood pressure among meat eaters, fish eaters, vegetarians and vegans in EPIC-Oxford. Public Health Nutr. 2002;5(5):645–54. 12372158 10.1079/PHN2002332

[86] Lee KW, Loh HC, Ching SM, Devaraj NK, Hoo FK. Effects of vegetarian diets on blood pressure lowering: a systematic review with Meta-Analysis and Trial Sequential Analysis. Nutrients 2020;12(6). 10.3390/nu12061604 PMC7352826 32486102

[87] Glenn AJ, Wang F, Tessier A-J, et al. Dietary plant-to-animal protein ratio and risk of cardiovascular disease in 3 prospective cohorts. Am J Clin Nutr. 2024;120(6):1373–86. 39631999 10.1016/j.ajcnut.2024.09.006 PMC12121410

[88] Strilchuk L, Cincione RI, Fogacci F, Cicero AFG. Dietary interventions in blood pressure lowering: current evidence in 2020. Kardiol Pol. 2020;78(7–8):659–66. 32631027 10.33963/KP.15468

[89] Sacks FM, Campos H. Dietary therapy in hypertension. N Engl J Med. 2010;362(22):2102–12. 20519681 10.1056/NEJMct0911013

[90] Willett W, Rockstrom J, Loken B, et al. Food in the Anthropocene: the EAT-Lancet Commission on healthy diets from sustainable food systems. Lancet. 2019;393(10170):447–92. 30660336 10.1016/S0140-6736(18)31788-4

[91] Sanders LM, Wilcox OMPML, Maki KC. Beef Consumption and Cardiovascular Risk factors: a systematic review and Meta-analysis of Randomized controlled trials, current developments in Nutrition. Curr Develop Nutr 2024;8(12):104500. 10.1016/j.cdnut.2024.104500 PMC11621491 39649475

[92] Services USDoAaUSDoHaH. Dietary Guidelines for Americans, 2020–2025. DietaryGuidelines.gov. Published 2020. Accessed November 7. 2024, 2024.

[93] Kirkpatrick CF, Sikand G, Petersen KS, et al. Nutrition interventions for adults with dyslipidemia: a clinical perspective from the National Lipid Association. J Clin Lipidol. 2023;17(4):428–51. 37271600 10.1016/j.jacl.2023.05.099

[94] Association AH. The American Heart Association Diet and Lifestyle Recommendations. 2021.

[95] Lloyd-Jones DM, Allen NB, Anderson CAM, et al. Life’s essential 8: updating and enhancing the American Heart Association’s construct of Cardiovascular Health: a Presidential Advisory from the American Heart Association. Circulation. 2022;146(5):e18–43. 35766027 10.1161/CIR.0000000000001078 PMC10503546

[96] Service NH. The Eatwell Guide. National Health Service. https://www.nhs.uk/live-well/eat-well/the-eatwell-guide/ Published 2023. Accessed November 7, 2024, 2024.

[97] Council NHaMR. A modelling system to inform the revision of the Australian Guide to Healthy Eating. https://www.health.gov.au/resources/publications/the-australian-dietary-guidelines?language=en. Published 2013. Updated 15 July 2019. Accessed November 7, 2024, 2024.

[98] Meinila J, Virtanen JK. Meat and meat products - a scoping review for Nordic Nutrition recommendations 2023. Food Nutr Res 2024;68. 10.29219/fnr.v68.10538 PMC10916397 38449706

[99] Health Mo. Eating and Activity Guidelines for New Zealand Adults: Updated 2020. Ministry of Health. https://www.tewhatuora.govt.nz/assets/For-the-health-sector/Health-sector-guidance/Active-Families/eating-activity-guidelines-new-zealand-adults-updated-2020-oct22.pdf. Published 2020. Accessed November 7, 2024, 2024.

[100] Bonekamp NE, Geleijnse JM, van der Schouw YT, et al. Dietary habits and compliance with dietary guidelines in patients with established cardiovascular disease. Eur J Clin Nutr. 2024;78(8):709–17. 38802604 10.1038/s41430-024-01443-7

[101] Wang Y, Beydoun MA, Caballero B, Gary TL, Lawrence R. Trends and correlates in meat consumption patterns in the US adult population. Public Health Nutr. 2010;13(9):1333–45. 20188005 10.1017/S1368980010000224 PMC2916052

